# An updated prevalence of asthma, its phenotypes, and the identification of the potential asthma risk factors among young Chinese adults recruited in Singapore

**DOI:** 10.1016/j.waojou.2023.100757

**Published:** 2023-03-20

**Authors:** Qi Yi Ambrose Wong, Jun Jie Lim, Jun Yan Ng, Praneeth Malipeddi, Yi Ying Eliza Lim, Yang Yie Sio, Fook Tim Chew

**Affiliations:** a Department of Biological Sciences, Faculty of Science, National University of Singapore, 117543, Singapore

**Keywords:** Asthma, Epidemiology, ISAAC, Prevalence, Risk factors

## Abstract

**Background:**

Asthma is a chronic inflammatory disease of the airway characterized by respiratory symptoms: wheezing, shortness of breath, coughing, and chest tightness. Globally, asthma affects over 300 million individuals and carries high morbidity and mortality burden. Previous studies have estimated the prevalence of asthma; however, prevalence estimates have been changing over time. Here, in a population of young Chinese adults from Singapore, we aimed to obtain an updated prevalence of asthma and its phenotypes, and identify potential associated risk factors.

**Methods:**

The Singapore/Malaysia Cross-Sectional Genetics Epidemiology Study (SMCGES) is an ongoing study which uses established ISAAC guidelines to collect epidemiological data and information pertaining to allergic diseases such as asthma. Responses from young Chinese adults recruited in the National University of Singapore were analyzed.

**Results:**

Lifetime asthma prevalence rate was estimated at 19.1% (2049/10,736), while current asthma prevalence rate was estimated at 6.3% (679/10,736). For ever asthma, the most important risk factor was a parental history of asthma. Increased consumption of pulses (aOR: 0.822, 95% CI: 0.706–0.958) was associated with a lowered odds of ever asthma, but cereals (aOR: 1.256, 95% CI: 1.006–1.580), pasta (aOR: 1.265, 95% CI: 1.027–1.553), butter (aOR: 1.350, 95% CI: 1.113–1.632), and margarine (aOR: 1.343, 95% CI: 1.081–1.660) were associated with a higher risk of ever asthma. Increased television/computer usage was associated with a decreased risk of ever asthma (aOR: 0.448, 95% CI: 0.367–0.545). Conversely, genetic factors had a lower strength of effect on current asthma (parental history of asthma - OR: 1.465, 95% CI: 1.135–1.888) as compared to ever asthma. Only increased potato consumption was significantly associated with an increased risk of current asthma (most or all days per week vs never or only occasionally - aOR: 1.577, 95% CI: 1.145–2.180). Physical activity (aOR: 0.693, 95% CI: 0.542–0.885) was associated with a lower odds of asthma, while second-hand smoke exposure was associated with an increased risk for current asthma (aOR: 1.435, 95% CI: 1.001–2.047).

**Conclusion:**

Overall, the prevalence of lifetime asthma and current asthma among young Chinese adults was 19.1% and 6.3%, higher than that of previous studies. Our results suggested a stronger association between genetic factors and ever asthma as compared to current asthma. Parental asthma was the most important intrinsic epidemiological factor for asthma manifestation, while various foods, physical activity levels, and television or computer usage were also significantly associated with asthma. Future studies should consider risk factors in conjunction with other accompanying variables given the potential interactions between them, to discern the effects of environment and lifestyle on asthma more distinctly.

## Introduction

### Background

Asthma is a chronic inflammatory disease of the airway characterized by respiratory symptoms: wheezing, shortness of breath, coughing, and chest tightness.^1^ Over 300 million individuals suffer from asthma worldwide, with an additional 100 million individuals projected to be at risk.^2^ Furthermore, asthma carries a significant morbidity and mortality burden, adversely affecting the quality of living and causing premature death, rendering a global health issue which cannot be ignored.^2^ Monitoring initiatives have been established and adopted worldwide, including the European Community Respiratory Health Survey (ECRHS), International Study of Asthma and Allergies in Childhood (ISAAC), and World Health Survey (WHS), providing constant worldwide updates on the prevalence and epidemiology of asthma.^3^, ^4^, ^5^ However, data from studies using standardized methods are often heterogeneous due variability between study populations, owing to demographic and geographic differences.^6^

### Objectives

Although the prevalence of asthma in Singapore has previously been reported, it has been established that asthma prevalence has been changing across time.^7^, ^8^, ^9^, ^10^, ^11^, ^12^ Presently, we investigate a sample of young Chinese adults in Singapore with 2 aims: (i) obtaining an updated prevalence of asthma and its phenotypes; and (ii) identifying the epidemiological factors associated with asthma using the ISAAC questionnaire.

## Materials and methods

### Participants, outcome definition, classification, and characterization

The Singapore/Malaysia Cohort Genetic Epidemiology Study (SMCGES) is an ongoing cross-sectional study conducted in Singapore and Malaysia. Since August 2005, participants were recruited via email and poster advertisements across the campuses of National University of Singapore, Sunway University, and Universiti Tunku Abdul Rahman for the SMCGES. Participants below the age of 18 were excluded. The current paper reports the data obtained from Singapore only; data from Malaysia will be analyzed and published separately.

Atopy status was determined via a Skin Prick Test (SPT), which tested for sensitization to allergens from the house dust mite species *Blomia tropicalis* and *Dermatophagoides pteronyssinus*. A positive histamine control and a negative saline control were included in the SPT. Subjects who developed a wheal of at least 3 mm in diameter in response to a given allergen were considered SPT positive or atopic. The SPT protocol was consistent with previous descriptions.^13^

Data for asthma were collected according to established and validated ISAAC guidelines which have been expanded for utility in both children and adults.^14^ Ever asthma cases comprised subjects who indicated ever having had asthma for the question: “Have you ever had asthma?”. Out of the ever asthma cases, those exhibiting a positive SPT result were further categorized as atopic asthma cases. Ever asthma cases were further classified as current asthma cases when any asthma symptoms within the past 12 months were reported – these included wheezing, dry coughing in the absence of a cold or flu, or any asthma exacerbations. Ever asthma subjects who manifested both a positive SPT result and any asthma symptoms within the past 12 months were hence considered current atopic asthma cases. Among current asthma cases, we further distinguished between exercise-induced asthma (experienced wheezy-sounding chest in the last 12 months) and cough-variant asthma (experienced a dry cough at night, which was not associated with a cold or chest infection in the last 12 months).

### Collection of epidemiological data

Epidemiological data collection was performed according to ISAAC protocol, yielding information of pertinence to demographics, familiar background, lifestyle, and diet.^14^

Basic demographic information concerning age, gender, income category, and housing type were collected. Additionally, the country of origin, years spent in Singapore for non-locals, and personal history of drug allergy were determined. To elucidate familiar and thus genetic predisposition, we identified those with a maternal, paternal, or sibling history of allergic diseases: atopic dermatitis, allergic rhinitis, and asthma. A general overview of participants’ lifestyles was obtained with regard to their physical activity levels (performed never or only occasionally, once or twice per week, or on most or all days), duration of television or computer usage (time spent per day was less than 1 h, 1 to 3 h, more than 3 but less than 5 h, or more than 5 h), alcohol consumption (never, occasionally, or frequently), and smoking status (non-smoker, ex-smoker, or current smoker). As an indicator of animal exposure, subjects indicated whether they had ever kept pets. Lastly, our dietary gathered information on 16 food groups – meat, seafood, fruits, vegetables, pulses, cereals, pasta, rice, butter, margarine, nuts, potato, milk, eggs, fast food (including burgers), and probiotic drinks. To each of these food groups, the frequency of their consumption by each subject was categorized within one of 3 options: never or only occasionally, once or twice per week, and most or all days.

### Scoring of overall glycemic index (GI) level of diet using the quality of diet based on Glycemic Index Score (QDGIS)

Using the Quality of Diet based on Glycemic Index Score (QDGIS), overall dietary glycemic index (GI) quality was assessed by scoring food consumption according to their glycemic index and intake frequency. "High-GI" foods had a GI value of 55 and above, and comprised burgers/fast food, cereals, rice, and potatoes; "low-GI" foods had a GI value of less than 55, and comprised fruits, vegetables, pulses, nuts, milk, and probiotic drinks.^15^^,^^16^ Next, we adapted a previously used rubric to quantify dietary GI.^17^ For each food, each category of consumption frequency was assigned scores accordingly: most or all days – score 7, once or twice per week – score 2, and never or only occasionally – score 0. Negative signs were prepended to scores for "high-GI" foods and positive signs were prepended to scores for "low-GI" foods, wherein increased consumption of "high-GI" foods resulted in a more negative score, while increased consumption of "low-GI" foods resulted in a higher positive score. The summation of all scores yielded the QDGIS which we then grouped into poor (QDGIS >2), moderate (2 ≤ QDGIS <10), and good (QDGIS ≥10) categories.

### Statistical analyses

Statistical analyses were conducted using R version 4.0.3.^18^ For analyses of epidemiological to identify associated factors, unadjusted odds ratios (OR) and their corresponding 95% confidence intervals (95% CI) were first calculated via simple logistic regression where the outcome of interest was ever having had asthma. Next, multiple logistic regression was conducted to adjust for important confounding variables, yielding adjusted odds ratios (aOR) and their respective 95% CI. To further elucidate the relationship between environmental variables and asthma, the logistic regression analyses were repeated, with the outcome being current asthma compared against non-current asthma cases. Effect sizes with a corresponding p-value of less than 0.05 were considered statistically significant.

## Results

### Sample demographics

Data from 10 736 participants of Chinese ethnicity from the Singapore cohort recruited from the National University of Singapore were analyzed. The mean age of sample subjects was 22.5 years (standard deviation (SD) = 5.2 years) and there was a preponderance of female subjects (57.6%). The most common total monthly family income per capita was SGD2000 to SGD4000 (34.0%), with many residing in public housing (67.5%). Majority were local Singaporean Chinese (63.1%) and within the non-local subgroup, the mean duration spent in Singapore was 5.9 years (SD = 6.8 years). A summary of sample demographics can be found in Table 1.

**Table 1:** Summary table for demographics of sample drawn from a population of young Chinese adult Singaporeans

Demographic factor	Summary
**Age (mean years ± standard deviation)**	22.5 ± 5.2
NA	60
**Gender**	
Female	6169 (57.6%)
Male	4545 (42.4%)
NA	22
**Total monthly family income per capita**	
< SGD2000	2350 (22.7%)
SGD2000 to < SGD4000	3528 (34.0%)
SGD 4000 to < SGD6000	2026 (19.5%)
≥ SGD6000	2466 (23.8%)
NA	366
**Housing type**	
HDB (Public housing)	6848 (67.5%)
Condominium/Private apartments	1928 (19.0%)
Landed property	1372 (13.5%)
NA	588
**Born in Singapore**	
Yes	7385 (69.1%)
No	3297 (30.9%)
NA	54
**Years spent in Singapore among non-locals (mean years ± standard deviation)**	5.9 ± 6.8
NA	262
**History of drug allergy**	
No	8847 (88.3%)
Yes	1170 (11.7%)
NA	719

### Prevalence of asthma and asthma phenotypes

**Fig. 1:**
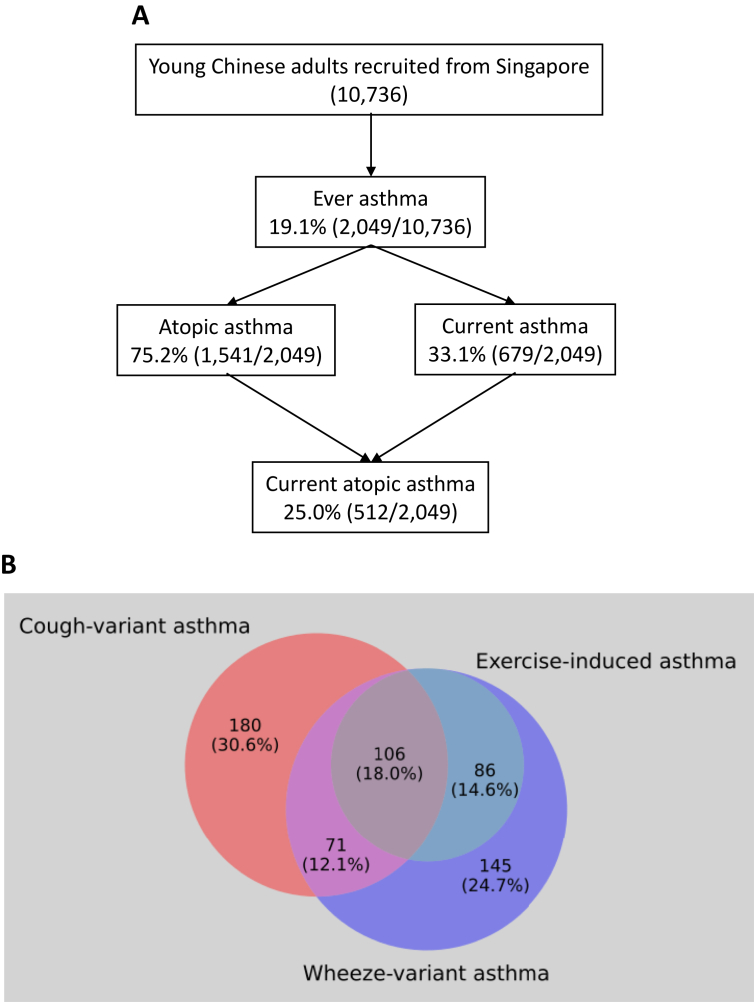
Summary of asthma phenotypes. (A) Flowchart summarizing participants' disease status; (B) Asthma variant distribution among current asthma cases with complete data for asthma variant symptoms (n = 588).

The prevalence of ever asthma was estimated at 19.1% (2049/10,736). Of the ever asthma cases, 75.2% (1541/2049) were atopic asthma cases and 33.1% (679/2049) were current asthma cases (Fig. 1A). Current atopic asthma cases constituted 25.0% (512/2049) of ever asthma cases. Among current asthma cases, 69.4% (408/588) exhibited symptoms of wheeze-variant asthma (WVA), and 47.1% (192/408) of WVA cases had exercise-induced asthma (EIA). 60.7% (357/588) of current asthma cases manifested cough-variant asthma. The asthma variants were non-mutually exclusive – 30.1% (177/588) had both CVA and WVA, while 18.0% (106/588) had all of CVA, EIA, and WVA. A breakdown of asthma variants is summarized in Fig. 1B.

### Epidemiological factors associated with ever asthma

#### Demographic factors

Demographic characteristics significantly associated with ever asthma included male gender was significantly associated with an increased odds of asthma (OR: 1.387, 95% CI: 1.255–1.532, p-value <0.001), a higher total monthly family income per capita which increased the odds of asthma in a dose-effect manner (≥SGD6000 vs < SGD2000 - OR: 1.536, 95% CI: 1.322–1.786, p-value <0.001), being born in Singapore (OR: 2.337, 95% CI: 2.071–2.643, p-value <0.001), and having a history of drug allergy (OR: 1.675, 95% CI: 1.448–1.935, p-value <0.001). Conversely, increased age (p-value = 0.2) and housing type (p-value = 0.864) were non-significantly associated with ever asthma.

Adjustment for gender and parental history of asthma showed that an increased odds of asthma was significantly associated with increased income levels in a dose-effect manner (aOR: 1.558, 95% CI: 1.299, 1.872, p-value <0.001), being born in Singapore (aOR: 2.334, 95% CI: 2.020, 2.706, p-value <0.001), and a history of drug allergy (aOR: 1.692, 95% CI: 1.419, 2.012, p-value <0.001). Increased age (p-value = 0.15) and housing type (p-value = 0.072) were non-significantly associated with ever asthma.

**Fig. 2:**
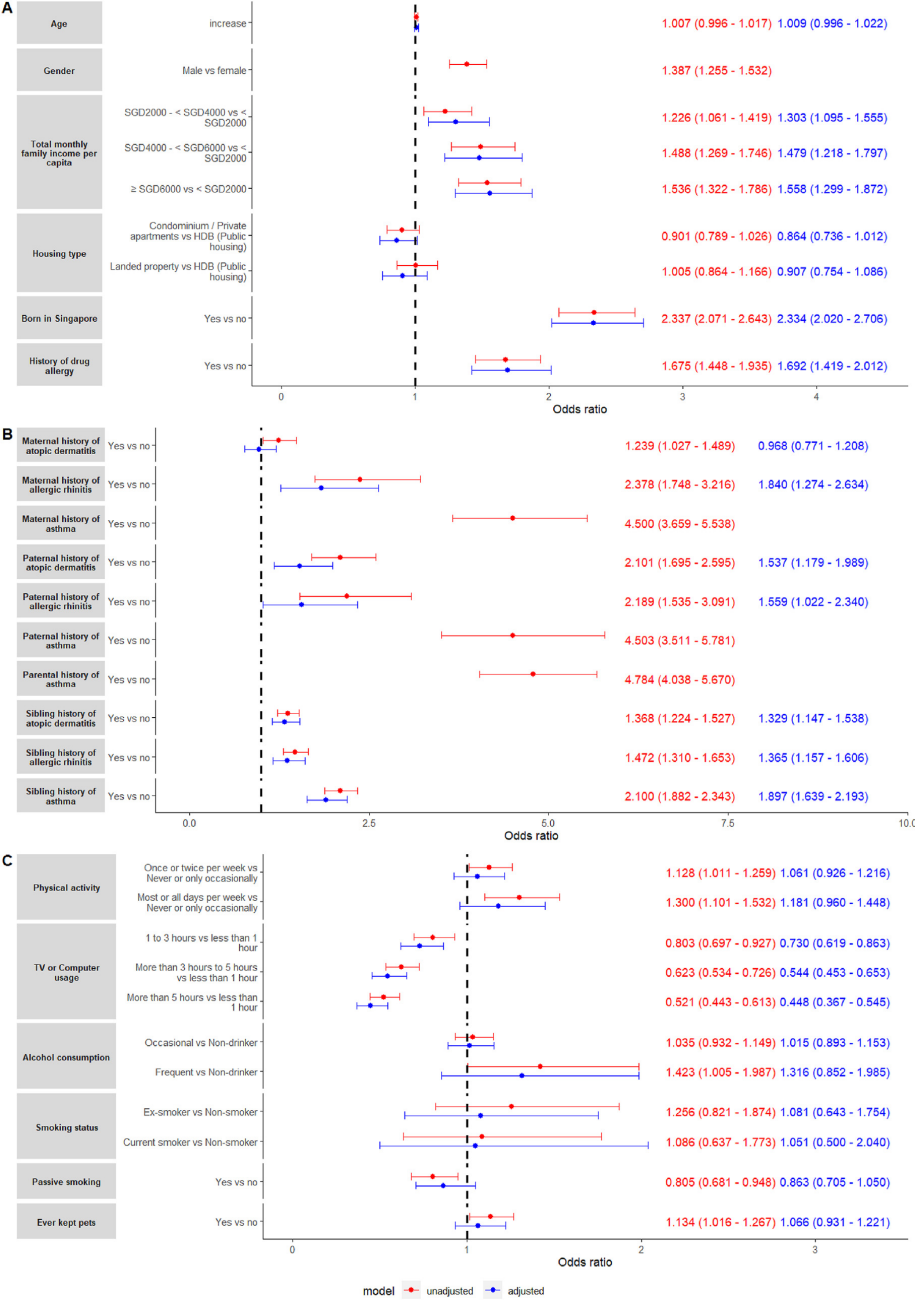
Forest plots for unadjusted (red text) and adjusted (blue text) odds ratios for ever asthma and potential risk factors. (A) Demographic characteristics; (B) Familiar history of allergic diseases; (C) Lifestyle habits.

A forest plot summarizing the associations between ever asthma and demographic factors can be found in Fig. 2A.

#### Familiar background

The odds of ever asthma were significantly increased given a maternal history of atopic dermatitis (OR: 1.239, 95% CI: 1.027–1.489, p-value = 0.023), allergic rhinitis (OR: 2.378, 95% CI: 1.748–3.216, p-value <0.001), and asthma (OR: 4.500, 95% CI: 3.659–5.538, p-value <0.001). Likewise, the odds of ever asthma were significantly increased given a paternal history of atopic dermatitis (OR: 2.101, 95% CI: 1.695–2.595, p-value <0.001), allergic rhinitis (OR: 2.189, 95% CI: 1.535–3.091, p-value <0.001), and asthma (OR: 4.503, 95% CI: 3.511–5.781, p-value <0.001). Overall, having any parental history of asthma significantly increased the odds of ever asthma (OR: 4.784, 95% CI: 4.038–5.670, p-value <0.001). Among those with siblings, the odds of ever asthma were significantly increased in those with a sibling history of atopic dermatitis (OR: 1.368, 95% CI: 1.224–1.527, p-value <0.001), allergic rhinitis (OR: 1.472, 95% CI: 1.310–1.653, p-value <0.001), or asthma (OR: 2.100, 95% CI: 1.882–2.343, p-value 0.017).

Following adjustment for gender and parental history of asthma, an increased odds of ever asthma was associated with maternal allergic rhinitis (aOR: 1.840, 95% CI: 1.274–2.634, p-value <0.001), paternal atopic dermatitis (aOR: 1.537, 95% CI: 1.179–1.989, p-value = 0.001), and paternal allergic rhinitis (aOR: 1.559, 95% CI: 1.022–2.340, p-value = 0.035), but not maternal atopic dermatitis (p-value = 0.8). An increased odds of ever asthma was significantly associated a sibling history of atopic dermatitis (aOR: 1.329, 95% CI: 1.147–1.538, p-value <0.001), allergic rhinitis (aOR: 1.365, 95% CI: 1.157–1.606, p-value <0.001), and asthma (aOR: 1.897, 95% CI: 1.639–2.193, p-value <0.001).

A forest plot summarizing the associations between ever asthma and familiar history of allergic diseases can be found in Fig. 2B.

#### Lifestyle

Statistically significant unadjusted associations between physical activity, television or computer usage, and alcohol consumption were found. An increased odds of ever asthma was associated with increased frequency of physical activity (most or all days per week vs never or only occasionally - OR: 1.300, 95% CI: 1.101–1.532, p-value = 0.002), frequent alcohol consumption (frequent vs never - OR: 1.423, 95% CI: 1.005–1.987, p-value = 0.042), and ever keeping pets (OR: 1.134, 95% CI: 1.016–1.267, p-value = 0.025). Increased computer usage was associated with a reduced odds of ever asthma (more than 5 h vs less than 1 h – OR: 0.521, 95% CI: 0.443–0.613, p-value <0.001), and there was dose-effect trend. The association between smoking and ever asthma was non-significant (p-value = 0.8).

Inclusion of gender and parental history of asthma in the logistic model showed that television or computer usage was significantly associated with a reduced odds of ever asthma (aOR: 0.448, 95% CI: 0.367–0.545, p-value <0.001). The associations between asthma and physical activity (p-value = 0.11), alcohol consumption (p-value = 0.2), smoking status (p-value = 0.9), and ever keeping pets (p-value = 0.4) were all non-significant.

A forest plot summarizing the associations between lifestyle habits and ever asthma is presented in Fig. 2C.

#### Diet

Unadjusted analyses showed that an increased odds of asthma was associated with increased seafood consumption (most or all days per week vs never or only occasionally - OR: 1.341, 95% CI: 1.094–1.655, p-value = 0.005), pasta (most or all days per week vs never or only occasionally - OR: 1.324, 95% CI: 1.119–1.564, p-value = 0.001), butter (most or all days per week vs never or only occasionally - OR: 1.356, 95% CI: 1.157–1.588, p-value <0.001), margarine (most or all days per week vs never or only occasionally - OR: 1.287, 95% CI: 1.075–1.536, p-value = 0.005), nuts (most or all days per week vs never or only occasionally - OR: 1.230, 95% CI: 1.023–1.475, p-value = 0.026), and potatoes (most or all days per week vs never or only occasionally - OR: 1.257, 95% CI: 1.058–1.494, p-value = 0.009).

Conversely, foods associated with a lower odds asthma included fruits (most or all days per week vs never or only occasionally - OR: 0.769, 95% CI: 0.609–0.977, p-value = 0.029), vegetables (most or all days per week vs never or only occasionally - OR: 0.732, 95% CI: 0.545–0.993, p-value = 0.041), pulses (once or twice per week vs never or only occasionally - OR: 0.803, 95% CI: 0.710–0.908, p-value = 0.001), and probiotic drinks (once or twice per week vs never or only occasionally - OR: 0.873, 95% CI: 0.784–0.972, p-value = 0.013). In consideration of overall diet, a moderate GI level compared to poor GI level associated with a lowered risk of asthma (OR: 0.831, 95% CI: 0.740–0.933, p-value = 0.002). However, while a similar protective association was observed in good GI level as compared to poor GI level, this was statistically non-significant (p-value = 0.2).

Upon adjustment for age and gender, we found that an increased odds of ever asthma was significantly associated with increased consumption of cereals (aOR: 1.256, 95% CI: 1.006–1.580, p-value = 0.047), pasta (aOR: 1.265, 95% CI: 1.027–1.553, p-value = 0.026), butter (aOR: 1.350, 95% CI: 1.113–1.632, p-value = 0.002), and margarine (aOR: 1.343, 95% CI: 1.081–1.660, p-value = 0.007). In contrast, pulses (aOR: 0.822, 95% CI: 0.706–0.958, p-value = 0.012) and probiotic drinks (aOR: 0.861, 95% CI: 0.756–0.980, p-value = 0.024) were associated with a lower odds of asthma. Overall, a moderate GI score compared to a poor GI score was significantly associated with an odds of ever asthma (aOR: 0.820, 95% CI: 0.712–0.944, p-value = 0.006).

The associations between ever asthma and demographic factors are summarized in Table 2.

**Table 2:** Summary of associations between dietary habits and ever asthma, including contingency tables with percentage for each response by ever asthma, and odds ratios, unadjusted and adjusted with their corresponding 95% confidence intervals as given by logistic regression models.

Dietary factor	N	Ever asthma		Univariate logistic regression			Multiple logistic regression^b^		
		No, N = 6,633^a^	Yes, N = 2,049^a^	Unadjusted OR^c^	95% CI^c^	p-value	Adjusted OR^c^	95% CI^c^	p-value
**Meat**	8656								
Never or only occasionally		162 (2.4%)	56 (2.7%)	–	–		–	–	
Once or twice per week		662 (10.0%)	181 (8.9%)	0.791	0.563, 1.124	0.2	0.745	0.492, 1.147	0.2
Most or all days per week		5791 (87.5%)	1804 (88.4%)	0.901	0.666, 1.236	0.5	0.864	0.599, 1.275	0.4
NA		18	8						
**Seafood**	8647								
Never or only occasionally		499 (7.6%)	129 (6.3%)	–	–		–	–	
Once or twice per week		3296 (49.9%)	936 (45.9%)	1.098	0.896, 1.355	0.4	0.998	0.785, 1.280	>0.9
Most or all days per week		2812 (42.6%)	975 (47.8%)	1.341	1.094, 1.655	0.005	1.238	0.974, 1.588	0.086
NA		26	9						
**Fruits**	8660								
Never or only occasionally		262 (4.0%)	104 (5.1%)	–	–		–	–	
Once or twice per week		2217 (33.5%)	679 (33.2%)	0.772	0.607, 0.987	0.036	0.805	0.593, 1.105	0.2
Most or all days per week		4136 (62.5%)	1262 (61.7%)	0.769	0.609, 0.977	0.029	0.803	0.597, 1.093	0.2
NA		18	4						
**Vegetables**	8628								
Never or only occasionally		149 (2.3%)	63 (3.1%)	–	–		–	–	
Once or twice per week		812 (12.3%)	235 (11.5%)	0.684	0.495, 0.955	0.024	0.748	0.496, 1.148	0.2
Most or all days per week		5628 (85.4%)	1741 (85.4%)	0.732	0.545, 0.993	0.041	0.798	0.549, 1.186	0.3
NA		44	10						
**Pulses**	8605								
Never or only occasionally		1397 (21.2%)	486 (23.9%)	–	–		–	–	
Once or twice per week		3929 (59.8%)	1097 (54.0%)	0.803	0.710, 0.908	<0.001	0.822	0.706, 0.958	0.012
Most or all days per week		1249 (19.0%)	447 (22.0%)	1.029	0.886, 1.194	0.7	1.037	0.862, 1.246	0.7
NA		58	19						
**Cereals**	8619								
Never or only occasionally		607 (9.2%)	170 (8.3%)	–	–		–	–	
Once or twice per week		2582 (39.2%)	759 (37.3%)	1.050	0.871, 1.270	0.6	1.122	0.892, 1.419	0.3
Most or all days per week		3393 (51.5%)	1108 (54.4%)	1.166	0.973, 1.404	0.10	1.256	1.006, 1.580	0.047
NA		51	12						
**Pasta**	8629								
Never or only occasionally		2430 (36.9%)	676 (33.2%)	–	–		–	–	
Once or twice per week		3473 (52.7%)	1110 (54.4%)	1.149	1.031, 1.281	0.012	1.217	1.067, 1.389	0.003
Most or all days per week		687 (10.4%)	253 (12.4%)	1.324	1.119, 1.564	0.001	1.265	1.027, 1.553	0.026
NA		43	10						
**Rice**	8624								
Never or only occasionally		141 (2.1%)	42 (2.1%)	–	–		–	–	
Once or twice per week		633 (9.6%)	207 (10.2%)	1.098	0.758, 1.618	0.6	0.986	0.631, 1.583	>0.9
Most or all days per week		5812 (88.2%)	1789 (87.8%)	1.033	0.736, 1.481	0.9	0.921	0.612, 1.431	0.7
NA		47	11						
**Butter**	8607								
Never or only occasionally		2895 (44.0%)	795 (39.2%)	–	–		–	–	
Once or twice per week		2937 (44.6%)	952 (47.0%)	1.180	1.060, 1.314	0.002	1.131	0.993, 1.289	0.064
Most or all days per week		749 (11.4%)	279 (13.8%)	1.356	1.157, 1.588	<0.001	1.350	1.113, 1.632	0.002
NA		52	23						
**Margarine**	8608								
Never or only occasionally		3621 (55.1%)	1033 (50.9%)	–	–		–	–	
Once or twice per week		2425 (36.9%)	803 (39.5%)	1.161	1.044, 1.290	0.006	1.155	1.015, 1.313	0.028
Most or all days per week		531 (8.1%)	195 (9.6%)	1.287	1.075, 1.536	0.005	1.343	1.081, 1.660	0.007
NA		56	18						
**Nuts**	8637								
Never or only occasionally		2837 (43.0%)	842 (41.3%)	–	–		–	–	
Once or twice per week		3238 (49.1%)	1006 (49.3%)	1.047	0.943, 1.162	0.4	1.047	0.922, 1.189	0.5
Most or all days per week		523 (7.9%)	191 (9.4%)	1.230	1.023, 1.475	0.026	1.235	0.988, 1.536	0.061
NA		35	10						
**Potatoes**	8632								
Never or only occasionally		1119 (17.0%)	325 (15.9%)	–	–		–	–	
Once or twice per week		4480 (68.0%)	1351 (66.3%)	1.038	0.906, 1.193	0.6	1.055	0.892, 1.253	0.5
Most or all days per week		994 (15.1%)	363 (17.8%)	1.257	1.058, 1.494	0.009	1.235	0.999, 1.529	0.051
NA		40	10						
**Milk**	8634								
Never or only occasionally		1349 (20.5%)	438 (21.5%)	–	–		–	–	
Once or twice per week		3072 (46.6%)	910 (44.6%)	0.912	0.801, 1.040	0.2	0.896	0.766, 1.050	0.2
Most or all days per week		2174 (33.0%)	691 (33.9%)	0.979	0.853, 1.124	0.8	0.933	0.790, 1.102	0.4
NA		38	10						
**Eggs**	8624								
Never or only occasionally		206 (3.1%)	67 (3.3%)	–	–		–	–	
Once or twice per week		2716 (41.2%)	840 (41.3%)	0.951	0.718, 1.274	0.7	1.084	0.761, 1.577	0.7
Most or all days per week		3666 (55.6%)	1129 (55.5%)	0.947	0.717, 1.266	0.7	1.011	0.711, 1.466	>0.9
NA		45	13						
**Burgers/fast food**	8625								
Never or only occasionally		2510 (38.1%)	765 (37.6%)	–	–		–	–	
Once or twice per week		3662 (55.6%)	1120 (55.0%)	1.003	0.904, 1.115	>0.9	1.005	0.885, 1.141	>0.9
Most or all days per week		418 (6.3%)	150 (7.4%)	1.177	0.959, 1.440	0.12	1.115	0.863, 1.430	0.4
NA		43	14						
**Probiotic drinks**	8624								
Never or only occasionally		2664 (40.4%)	889 (43.6%)	–	–		–	–	
Once or twice per week		2938 (44.6%)	856 (42.0%)	0.873	0.784, 0.972	0.013	0.861	0.756, 0.980	0.024
Most or all days per week		985 (15.0%)	292 (14.3%)	0.888	0.763, 1.032	0.12	0.877	0.729, 1.052	0.2
NA		46	12						
**GI level score (categorized)**	8411								
Poor		2135 (33.3%)	731 (36.7%)	–	–		–	–	
Moderate		2731 (42.6%)	777 (39.0%)	0.831	0.740, 0.933	0.002	0.820	0.712, 0.944	0.006
Good		1552 (24.2%)	485 (24.3%)	0.913	0.799, 1.041	0.2	0.913	0.777, 1.071	0.3
NA		215	56						

a n (%); Percentages were calculated column-wise.

b Adjusted for gender and parental history of asthma.

c OR: odds ratio; 95% CI: 95% confidence interval

### Epidemiological factors associated with current asthma

#### Demographic factors

Increased age (OR: 1.022, 95% CI: 1.002–1.041, p-value = 0.028) and female gender (OR: 0.764, 95% CI: 0.635–0.919, p-value = 0.004) were significantly associated with an increased odds of current asthma. Conversely, current asthma was not significantly associated with total monthly family income per capita (p-value = 0.5), housing type (p-value = 0.13), being born in Singapore (p-value = 0.4), and any history of drug allergy (p-value = 0.3).

Adjustment for gender and parental history of asthma showed that only age was significantly associated with and increased odds of current asthma (aOR: 1.023, 95% CI: 1.001–1.046, p-value = 0.039). There was no significant association between the odds of current asthma and total monthly family income per capita (p-value = 0.3), housing type (p-value = 0.092), being born in Singapore (p-value = 0.5), and any history of drug allergy (p-value = 0.3).

**Fig. 3:**
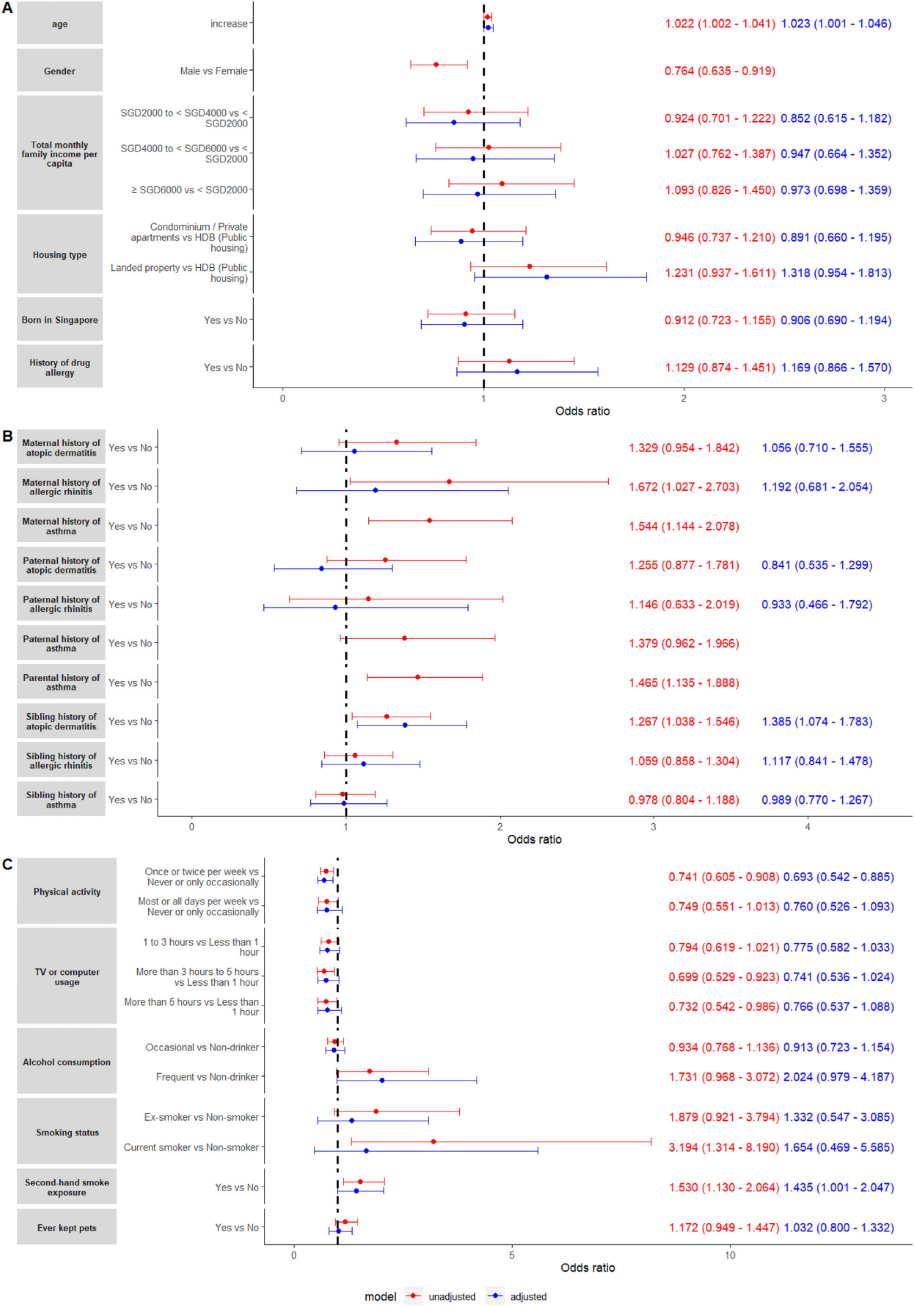
Forest plots for unadjusted (red text) and adjusted (blue text) odds ratios for current asthma and potential risk factors. (A) Demographic characteristics; (B) Familiar history of allergic diseases; (C) Lifestyle habits.

The associations between demographic factors and current asthma are summarized in a forest plot in Fig. 3A.

#### Familiar background

While a maternal history of atopic dermatitis (p-value = 0.089) was non-significantly associated with current asthma, a maternal history of allergic rhinitis (OR: 1.672, 95% CI: 1.027–2.703, p-value = 0.036) and asthma (OR: 1.544, 95% CI: 1.144–2.078, p-value = 0.004) were both associated with an increased odds of current asthma. In contrast, a paternal history of atopic dermatitis (p-value = 0.2), allergic rhinitis (p-value = 0.6), and asthma (p-value = 0.078) were all non-significantly associated with current asthma. Overall, any parental history of asthma was significantly associated with an increased odds of current asthma (OR: 1.465, 95% CI: 1.135–1.888, p-value = 0.003). A sibling history of allergic rhinitis (p-value = 0.6), and a sibling history of asthma (p-value = 0.8) were not significantly associated with current asthma. However, a sibling history of atopic dermatitis (OR: 1.267, 95% CI: 1.038–1.546, p-value = 0.020) was significantly associated with an increased odds of current asthma.

Upon adjustment for gender and parental history of asthma, a maternal history of atopic dermatitis (p-value = 0.8) and allergic rhinitis (p-value = 0.5), and a paternal history of atopic dermatitis (p-value = 0.4) and allergic rhinitis (p-value = 0.8) all were all non-significantly associated with the odds of current asthma. A sibling history of atopic dermatitis was significantly associated with an increased odds of current asthma (aOR: 1.385, 95% CI: 1.074–1.783, p-value = 0.012). Having siblings (p-value = 0.8), a sibling history of allergic rhinitis (p-value = 0.4), and a sibling history of asthma (p-value >0.9) were all non-significantly associated with current asthma.

The associations between current asthma and familiar history are summarized in Fig. 3B.

#### Lifestyle and dietary habits

Increased physical activity was significantly associated with a decreased odds of current asthma (once or twice per week vs never or only occasionally - OR: 0.741 95% CI: 0.605–0.908, p-value = 0.004), but this association was not observed at higher frequencies of physical activity (most or all days per week vs never or only occasionally - OR: 0.749, 95% CI: 0.551–1.013, p-value = 0.063). An average television or computer usage time of more than 3 h was significantly associated with a decreased odds of current asthma (more than 3 h–5 h vs less than 1 h – OR: 0.699, 95% CI: 0.529–0.923, p-value = 0.012; more than 5 h vs less than 1 h – OR: 0.732, 95% CI: 0.542–0.986, p-value = 0.041). Conversely, current smoking (OR: 3.194, 95% CI: 1.314–8.190, p-value = 0.011) and exposure to second-hand smoke (OR: 1.530, 95% CI: 1.130–2.064, p-value = 0.006) were significantly associated with an increased odds of current asthma. However, frequent alcohol consumption (p-value = 0.061), and ever keeping pets (p-value = 0.14) were non-significantly associated with current asthma. Fig. 3C summarizes the associations between lifestyle habits and current asthma.

Of 16 food items, only increased potato consumption was significantly associated with an increased odds of current asthma (once or twice per week vs never or only occasionally - aOR: 1.428, 95% CI: 1.034–1.993, p-value = 0.033), wherein a dose-effect trend was also observed (most or all days per week vs never or only occasionally - aOR: 1.577, 95% CI: 1.145–2.180, p-value = 0.006). All other foods were non-significantly associated with current asthma (see Table 3).

**Table 3:** Summary of associations between dietary habits and current asthma, including contingency tables with percentage for each response by ever asthma, and odds ratios, unadjusted and adjusted with their corresponding 95% confidence intervals as given by logistic regression models.

Characteristic	N	Current asthma		Univariate logistic regression			Multiple logistic regression^b^		
		No, N = 1,370^a^	Yes, N = 679^a^	Unadjusted OR^c^	95% CI^c^	p-value	Adjusted OR^c^	95% CI^c^	p-value
**Meat**	2041								
Never or only occasionally		39 (2.9%)	17 (2.5%)	–	–		–	–	
Once or twice per week		130 (9.5%)	51 (7.5%)	0.900	0.473, 1.764	0.8	0.969	0.449, 2.177	>0.9
Most or all days per week		1196 (87.6%)	608 (89.9%)	1.166	0.665, 2.131	0.6	1.168	0.594, 2.428	0.7
NA		5	3						
**Seafood**	2040								
Never or only occasionally		88 (6.5%)	41 (6.1%)	–	–		–	–	
Once or twice per week		623 (45.7%)	313 (46.3%)	1.078	0.731, 1.613	0.7	1.056	0.677, 1.674	0.8
Most or all days per week		653 (47.9%)	322 (47.6%)	1.058	0.718, 1.582	0.8	1.029	0.660, 1.630	>0.9
NA		6	3						
**Fruits**	2045								
Never or only occasionally		73 (5.3%)	31 (4.6%)	–	–		–	–	
Once or twice per week		456 (33.3%)	223 (32.9%)	1.152	0.741, 1.827	0.5	1.324	0.747, 2.449	0.4
Most or all days per week		839 (61.3%)	423 (62.5%)	1.187	0.775, 1.860	0.4	1.459	0.837, 2.659	0.2
NA		2	2						
**Vegetables**	2039								
Never or only occasionally		43 (3.2%)	20 (3.0%)	–	–		–	–	
Once or twice per week		153 (11.2%)	82 (12.1%)	1.152	0.643, 2.121	0.6	1.512	0.698, 3.500	0.3
Most or all days per week		1167 (85.6%)	574 (84.9%)	1.057	0.625, 1.851	0.8	1.491	0.738, 3.266	0.3
NA		7	3						
**Pulses**	2030								
Never or only occasionally		323 (23.8%)	163 (24.3%)	–	–		–	–	
Once or twice per week		741 (54.5%)	356 (53.1%)	0.952	0.760, 1.196	0.7	0.961	0.731, 1.267	0.8
Most or all days per week		295 (21.7%)	152 (22.7%)	1.021	0.778, 1.340	0.9	0.916	0.657, 1.276	0.6
NA		11	8						
**Cereals**	2037								
Never or only occasionally		107 (7.9%)	63 (9.3%)	–	–		–	–	
Once or twice per week		512 (37.6%)	247 (36.5%)	0.819	0.581, 1.163	0.3	0.971	0.641, 1.485	0.9
Most or all days per week		742 (54.5%)	366 (54.1%)	0.838	0.601, 1.176	0.3	0.879	0.587, 1.330	0.5
NA		9	3						
**Pasta**	2039								
Never or only occasionally		453 (33.2%)	223 (33.1%)	–	–		–	–	
Once or twice per week		746 (54.7%)	364 (54.0%)	0.991	0.809, 1.216	>0.9	0.947	0.744, 1.207	0.7
Most or all days per week		166 (12.2%)	87 (12.9%)	1.065	0.783, 1.442	0.7	0.986	0.677, 1.426	>0.9
NA		5	5						
**Rice**	2038								
Never or only occasionally		26 (1.9%)	16 (2.4%)	–	–		–	–	
Once or twice per week		132 (9.7%)	75 (11.1%)	0.923	0.470, 1.860	0.8	0.976	0.433, 2.283	>0.9
Most or all days per week		1206 (88.4%)	583 (86.5%)	0.786	0.422, 1.506	0.5	0.870	0.411, 1.927	0.7
NA		6	5						
**Butter**	2026								
Never or only occasionally		526 (38.7%)	269 (40.3%)	–	–		–	–	
Once or twice per week		645 (47.5%)	307 (46.0%)	0.931	0.762, 1.137	0.5	0.869	0.685, 1.102	0.2
Most or all days per week		187 (13.8%)	92 (13.8%)	0.962	0.718, 1.282	0.8	0.917	0.648, 1.289	0.6
NA		12	11						
**Margarine**	2031								
Never or only occasionally		675 (49.7%)	358 (53.3%)	–	–		–	–	
Once or twice per week		550 (40.5%)	253 (37.6%)	0.867	0.712, 1.055	0.2	0.871	0.689, 1.101	0.2
Most or all days per week		134 (9.9%)	61 (9.1%)	0.858	0.615, 1.187	0.4	0.868	0.587, 1.270	0.5
NA		11	7						
**Nuts**	2039								
Never or only occasionally		558 (40.9%)	284 (42.0%)	–	–		–	–	
Once or twice per week		672 (49.3%)	334 (49.4%)	0.977	0.804, 1.186	0.8	0.954	0.758, 1.203	0.7
Most or all days per week		133 (9.8%)	58 (8.6%)	0.857	0.607, 1.198	0.4	0.773	0.512, 1.152	0.2
NA		7	3						
**Potatoes**	2039								
Never or only occasionally		234 (17.2%)	91 (13.4%)	–	–		–	–	
Once or twice per week		903 (66.3%)	448 (66.2%)	1.276	0.980, 1.673	0.074	1.428	1.034, 1.993	0.033
Most or all days per week		225 (16.5%)	138 (20.4%)	1.577	1.145, 2.180	0.006	1.826	1.233, 2.718	0.003
NA		8	2						
**Milk**	2039								
Never or only occasionally		289 (21.2%)	149 (22.0%)	–	–		–	–	
Once or twice per week		632 (46.4%)	278 (41.1%)	0.853	0.670, 1.089	0.2	0.798	0.600, 1.063	0.12
Most or all days per week		442 (32.4%)	249 (36.8%)	1.093	0.850, 1.406	0.5	1.001	0.745, 1.348	>0.9
NA		7	3						
**Eggs**	2036								
Never or only occasionally		45 (3.3%)	22 (3.3%)	–	–		–	–	
Once or twice per week		559 (41.0%)	281 (41.7%)	1.028	0.612, 1.775	>0.9	1.935	0.961, 4.236	0.078
Most or all days per week		758 (55.7%)	371 (55.0%)	1.001	0.599, 1.721	>0.9	1.807	0.900, 3.947	0.11
NA		8	5						
**Burgers/fast food**	2035								
Never or only occasionally		519 (38.2%)	246 (36.3%)	–	–		–	–	
Once or twice per week		736 (54.2%)	384 (56.7%)	1.101	0.906, 1.339	0.3	1.194	0.947, 1.509	0.14
Most or all days per week		103 (7.6%)	47 (6.9%)	0.963	0.656, 1.396	0.8	0.961	0.598, 1.515	0.9
NA		12	2						
**Probiotic drinks**	2037								
Never or only occasionally		588 (43.2%)	301 (44.6%)	–	–		–	–	
Once or twice per week		581 (42.7%)	275 (40.7%)	0.925	0.757, 1.129	0.4	0.971	0.766, 1.230	0.8
Most or all days per week		193 (14.2%)	99 (14.7%)	1.002	0.756, 1.322	>0.9	1.073	0.768, 1.493	0.7
NA		8	4						
**GI level score (categorized)**	1993								
Poor		494 (37.1%)	237 (35.9%)	–	–		–	–	
Moderate		526 (39.5%)	251 (38.0%)	0.995	0.802, 1.234	>0.9	1.144	0.884, 1.482	0.3
Good		313 (23.5%)	172 (26.1%)	1.145	0.899, 1.459	0.3	1.182	0.883, 1.580	0.3
NA		37	19						

a n (%); Percentages were calculated column-wise.

b Adjusted for gender and parental history of asthma.

c OR: odds ratio; 95% CI: 95% confidence interval

Odds ratios adjusted for gender and parental history of asthma showed that physical activity was a significantly associated with a reduced likelihood of current asthma (aOR: 0.693, 95% CI: 0.542–0.885, p-value = 0.003), while exposure to second-hand smoke was significantly associated with current asthma (aOR: 1.435, 95% CI: 1.001–2.047, p-value = 0.047). Lifestyle habits non-significantly associated with the odds of current asthma included a longer duration of television or computer usage (p-value = 0.069), frequent alcohol consumption (p-value = 0.055), current smoking (p-value = 0.4), and ever keeping pets (p-value = 0.8).

## Discussion

From our cross-sectional study, we estimated a lifetime asthma prevalence rate of 19.1%, and a current asthma prevalence rate of 6.3%. In comparison, estimates for prevalence rate of lifetime asthma and current asthma were given by the Singapore Mental Health Study in 2016 as 11.9% and 2.6%, respectively, and by the National Health Survey as 10.5% and 3.9%, respectively.^11^^,^^19^ The present findings indicate a possible rise in lifetime asthma prevalence rate, as has been highlighted previously.^11^ The upward trend in asthma prevalence mirrors that which has been observed and reported worldwide, and reasoning for such patterns have been the subject of speculation.^10^ Nonetheless, hypotheses for the change in prevalence rate include a possible improvement in awareness of asthma, improvements in diagnosis, and better access to healthcare – all of which do not warrant immediate concern.^8^^,^^20^ Notwithstanding, changes in environment and increased exposure to potential risk factors present a distinct possibility for the cause of increasing asthma prevalence rates. Herein, we examined the environmental factors associated with both ever asthma and current asthma.

Overall, a comparison of factors affecting ever asthma and current asthma indicated that demographics played a significant role in the manifestation of lifetime asthma, but not in current asthma. Notably, age was non-significantly associated with ever asthma but significantly associated with a higher likelihood of current asthma; male gender, while associated with an increased odds of ever asthma, was contrastively associated with a lowered risk of current asthma. Age and gender differences in asthma have been recognized in the literature, where findings revealed that asthma diagnosis peaked in young males but in older women, while severe asthma and asthma exacerbations affected younger boys but older women.^21^^,^^22^ Potential reasons given for the observed discrepancy include the influence of sex hormones and difference in perception of asthma among women, who may respond distinctly to asthma manifestation.^22^^,^^23^ Concordantly, from our cohort of young Chinese adults, we provide further evidence of an age-gender interaction effect on asthma.

Additionally, the demographic characteristics of increased income, being born in Singapore, and a history of drug allergy were significantly associated with ever asthma, suggesting a role for these non-modifiable factors in the manifestation of lifetime asthma. Of interest, the association of income with asthma has been reported in the literature.^6^ While our findings for increased income as a significant factor for the likelihood of asthma corresponds to that of previous scientific literature, we found that the direction of association was discordant with some earlier reports – the cross-sectional National Health and Nutrition Examination Survey reported that family income below the poverty threshold was associated with an increased likelihood of asthma, and the longitudinal Western Australian Pregnancy Cohort (Raine) Study found that children from low-income households had a two-fold increased risk of asthma while increasing income levels was associated with a decreased risk of asthma.^24^^,^^25^ We posit that higher familiar income affords greater access to healthcare, leading to a higher rate of doctor visits and thus diagnosis of asthma, resulting in a greater prevalence of asthma among individuals from higher income families.^26^

A familiar history of atopic diseases (ie, atopic dermatitis, allergic rhinitis, and asthma) was significantly associated with an increased likelihood of ever asthma, but to a much lesser extent with current asthma. Notably, both unadjusted and adjusted odds ratios indicated that maternal and paternal allergic rhinitis, maternal and paternal asthma, and sibling diagnoses of atopic diseases were associated with an increased risk of asthma. Moreover, the crude effect sizes for ever asthma and the respective parental risk factors of maternal and paternal asthma were significantly high, with an odds ratio of at least 4.5. In contrast, the odds ratios for current asthma and the respective risk factors of maternal and paternal asthma were relatively low (OR < 2.0). Upon adjustment, a familiar history of allergic diseases was significantly associated with ever asthma, but not with current asthma. Findings from our earlier meta-analysis were consistent with our current findings, wherein familiar medical history was frequently associated with asthma manifestation, of which familiar history of asthma showed the significance in association.^6^ Indeed, the genetic influence of asthma has long since been identified, and heritability of asthma has been estimated to be as high as 95%; numerous candidate genes have been hitherto identified for asthma.^27^, ^28^, ^29^ Here, we further suggest that while genetics constitute an important risk factor in the manifestation of asthma, the persistence of ever asthma into current asthma sees their importance as risk factors diminish.

We have also examined the associations of selected lifestyle habits with both ever asthma, and current asthma. Adjusted odds ratios showed that only increased television or computer usage was significantly associated a decreased odds of ever asthma, while increased physical activity and exposure to second-hand smoke were significant risk factors for current asthma. Concerning physical activity, our findings once again contribute to a repository of inconsistent associations in the literature – higher physical activity has been variously associated with increased risk of asthma in some reports, and a decreased risk of asthma in others.^30^ However recent findings have indicated a potential link between sedentary lifestyle and asthma risk: a high screen time and low frequency of physical activity increased the risk of central obesity, which in turn correlated highly to the manifestation of asthma.^31^ We theorize that physical activity is merely a constituent to a multifactorial lifestyle habit component and interacts with other variables including screen time and duration to sleep, influencing a downstream manifestation of asthma.^30^^,^^32^ Moreover, the intensity and energy expenditure of physical activity need to be considered as well.^32^ With regards to second-hand smoke exposure, we note that while second-hand smoke exposure increased the odds of current asthma, smoking status itself was not significantly associated with asthma, likely due to the low proportion of smokers in our sample. Nonetheless, our findings for second-hand tobacco smoke exposure were concordant with that of previous studies, and reinforced the role of tobacco in increasing the risk of current asthma manifestation.^6^

Finally, we hypothesized that in addition to environmental factors, dietary habits play some role in the extension of ever asthma into current asthma: increased consumption of selected food groups - pulses, probiotic drinks, and a lower overall GI of diet were associated with a lower likelihood of ever asthma; cereals, pasta, butter, margarine, and potatoes were associated with an increased odds of ever asthma, while only potatoes were identified as a significant risk factor for current asthma. Interestingly, increased consumption of potatoes was associated with an increased likelihood of both ever asthma and current asthma in our cohort – to our knowledge an association hardly reported before. Our current results adds on to the numerous conflicting associations between food and asthma identified hitherto in the scientific literature.^33^^,^^34^ Among these, fruits, vegetables, meat, fish, and fast-food have been the most frequent food-asthma associations highlighted from ISAAC studies.^34^ Additional grouping of food types into dietary habits, such as "Mediterranean diet" or "Western diet" patterns, yielded no further conclusive associations.^34^ Moreover, our novel assessment of overall dietary GI, while associated with lowered likelihood of ever asthma in moderate GI level vs poor GI levels groups, showed no dose-effect relationship, and was non-significantly associated with current asthma. Nonetheless, the possible effects of foods synergies make dietary habits are a complex entity worth continued exploration.^35^ Since dietary habits have potentially distinct effects from food types in isolation, further investigations into food-asthma associations might find sagacity in the usage of principal component analysis or factor analysis methods to discern dietary patterns.^36^, ^37^, ^38^

## Limitations and conclusions

The cross-sectional nature of our study entailed the limitations inherent in cross-sectional studies. Findings for the relationships between potential risk factors and asthma were restricted to associations, while time-trend data could not be gathered from this study. In particular, we acknowledge the temporal factor in the relationship between dietary intake and asthma manifestation, which this study yielded insufficient data on. A longitudinal study design would better account for the possible influence of time in the association between diet and asthma. notwithstanding, the current study provides a starting point for further investigation, highlighting the important risk factors for ever asthma and current asthma. Upcoming genome-wide association studies (GWAS), expression quantitative trait loci (eQTL) analyses, and functional characterization studies would further elucidate the mechanisms involved in asthma manifestation and its associated risk factors.

In conclusion, we have provided an update to the prevalence of ever asthma, current asthma, and asthma phenotypes in a sample of young Chinese adults. As has been the case internationally, the prevalence of asthma has seen an increase in Singapore. Moreover, we have identified several risk factors of interest, such as familiar history of atopic diseases for ever asthma, and age, gender, dietary and lifestyle factors for current asthma. Overall, genetic factors appeared to have more importance in influencing ever asthma, while lifestyle and environmental factors may play a more dominant role in current asthma manifestation. Importantly, we realized that the risk factors should also be analyzed in combinations with other risk factors to account for potential interactions, and future studies including GWAS, eQTL analyses, and functional characterization could better elucidate mechanisms involved.

## Abbreviations

95% CI, 95% confidence interval; aOR, adjusted odds ratio; eQTL:, expression quantitative trait loci; GWAS, genome-wide association studies; ISAAC, International Study of Asthma and Allergies in Childhood; OR, odds ratio; SPT, skin prick test; TV, television.

## Acknowledgements

We extend our sincerest gratitude to all participants for their contributions to this study.

## Funding

F.T.C. received grants from the 10.13039/501100001352National University of Singapore (N-154-000-038-001), Singapore Ministry of Education Academic Research Fund (R-154-000-191-112; R-154-000-404-112; R-154-000-553-112; R-154-000-565-112; R-154-000-630-112; R-154-000-A08-592; R-154-000-A27-597; R-154-000-A91-592; R-154-000-A95-592; R154-000-B99-114), 10.13039/501100012415Biomedical Research Council (10.13039/501100012415BMRC) (Singapore) (10.13039/501100012415BMRC/01/1/21/18/077; 10.13039/501100012415BMRC/04/1/21/19/315; 10.13039/501100012415BMRC/APG2013/108), Singapore Immunology Network (SIgN-06-006; SIgN-08-020), 10.13039/501100001349National Medical Research Council (10.13039/501100001349NMRC) (Singapore) (10.13039/501100001349NMRC/1150/2008), 10.13039/501100001321National Research Foundation (10.13039/501100001381NRF) (Singapore) (NRF-MP-2020-0004), Singapore Food Agency (SFA) (SFS_RND_SUFP_001_04; W22W3D0006), and the Agency for Science Technology and Research (A∗STAR) (Singapore) (H17/01/a0/008; and APG2013/108). The funding agencies had no role in the study design, data collection and analysis, decision to publish, or preparation of the manuscript.

## Availability of data and materials

All data used and included in this study are available from the corresponding author (F.T.C.).

## Author contributions

F.T.C. conceived and supervised the current research study. Q.Y.A.W. conducted the literature review, analyzed and interpreted the data, and wrote the manuscript. Q.Y.A.W., J.J.L., J.Y.N., P.M., Y.Y.E.L., and Y.Y.S assisted in recruiting study participants and data collation. All authors read and approved the final manuscript.

## Authors’ consent for publication

All authors have read and consented to the publication of this manuscript.

## Ethics approval and consent

Ethical approval for this study was granted by the 10.13039/501100001352NUS Institutional Review Board (IRB reference code: NUS-07-023, NUS-09-256, NUS-10-445, NUS-13-075, NUS-14-150, and NUS-18-036). This study was performed in compliance with the Declaration of Helsinki, Good Clinical Practice, and local regulatory guidelines. Before participation, each subject was informed of this study's details via a Participant Information Sheet and provided written informed consent to participation through the signature of a Consent Form.

## Declaration of competing interest

F.T.C. reports grants from Singapore Ministry of Education Academic Research Fund, Singapore Immunology Network, National Medical Research Council (Singapore), Biomedical Research Council (Singapore), National Research Foundation (NRF) (Singapore), Singapore Food Agency (SFA), and the Agency for Science Technology and Research (Singapore), during the conduct of the study; and has received consultancy fees from Sime Darby Technology Centre, First Resources Ltd, Genting Plantation, Olam International, and Syngenta Crop Protection, outside the submitted work. The other authors declare no other competing interests.
